# Arsenic trioxide induces cell cycle arrest and affects Trk receptor expression in human neuroblastoma SK-N-SH cells

**DOI:** 10.1186/s40659-018-0167-6

**Published:** 2018-06-13

**Authors:** Xilin Xiong, Yang Li, Ling Liu, Kai Qi, Chi Zhang, Yueqin Chen, Jianpei Fang

**Affiliations:** 1 Guangdong Provincial Key Laboratory of Malignant Tumor Epigenetics and Gene Regulation, Sun Yat-Sen Memorial Hospital, Sun Yat-Sen University, Guangzhou, 510120 China; 2 Pediatric Hematology/Oncology, Sun Yat-Sen Memorial Hospital, Sun Yat-Sen University, Guangzhou, 510120 China; 3 Department of Pediatric Hematology/Oncology, Affiliated Hospital of Guangdong Medical College, Zhanjiang, 524000 Guangdong China; 4 Department of Life Sciences, Sun Yat-Sen University, Guangzhou, 510120 Guangdong China

**Keywords:** Arsenic trioxide, Neuroblastoma, Cell cycle, Trk expression

## Abstract

**Background:**

Arsenic trioxide (As_2_O_3_), a drug that has been used in China for approximately two thousand years, induces cell death in a variety of cancer cell types, including neuroblastoma (NB). The tyrosine kinase receptor (Trk) family comprises three members, namely TrkA, TrkB and TrkC. Various studies have confirmed that TrkA and TrkC expression is associated with a good prognosis in NB, while TrkB overexpression can lead to tumor cell growth and invasive metastasis. Previous studies have shown that As_2_O_3_ can inhibit the growth and proliferation of a human NB cell line and can also affect the N-Myc mRNA expression. It remains unclear whether As_2_O_3_ regulates Trks for the purposes of treating NB.

**Methods:**

The aim of the present study was to investigate the effect of As_2_O_3_ on Trk expression in NB cell lines and its potential therapeutic efficacy. SK-N-SH cells were grown with increasing doses of As_2_O_3_ at different time points. We cultured SK-N-SH cells, which were treated with increasing doses of As_2_O_3_ at different time points. Trk expression in the NB samples was quantified by immunohistochemistry, and the cell cycle was analyzed by flow cytometry. TrkA, TrkB and TrkC mRNA expression was evaluated by real-time PCR analysis.

**Results:**

Immunohistochemical and real-time PCR analyses indicated that TrkA and TrkC were over-expressed in NB, and specifically during stages 1, 2 and 4S of the disease progression. TrkB expression was increased in stage 3 and 4 NB. As_2_O_3_ significantly arrested SK-N-SH cells in the G2/M phase. In addition, TrkA, TrkB and TrkC expression levels were significantly upregulated by higher concentrations of As_2_O_3_ treatment, notably in the 48-h treatment period. Our findings suggested that to achieve the maximum effect and appropriate regulation of Trk expression in NB stages 1, 2 and 4S, As_2_O_3_ treatment should be at relatively higher concentrations for longer delivery times;however, for NB stages 3 and 4, an appropriate concentration and infusion time for As_2_O_3_ must be carefully determined.

**Conclusion:**

The present findings suggested that As_2_O_3_ induced Trk expression in SK-N-SH cells to varying degrees and may be a promising adjuvant to current treatments for NB due to its apoptotic effects.

## Background

Neuroblastoma (NB) is an extracranial solid tumor that is frequently encountered in children and accounts for 8–10% of malignancies [1]. Despite the use of intensive chemotherapy, the 5-year event-free survival is 30%, and remains unsatisfactory in the high-risk and refractory NB patient subgroup [2]. The main treatment for NB includes combined chemotherapy, radiotherapy and surgery. However, despite improvements in disease progression via intensification of therapy and efforts to treat minimal residual disease, more than 50% of patients will relapse and die from their malignancies [3]. Relapsed or refractory NB responds poorly to conventional treatments and has a lower survival rate [4]. In recent years, new therapies, such as auto-hematopoietic stem cell transplantation (auto-HSCT), targeting and biological immunotherapy treatment, have been used somewhat effectively in patients with relapsed or refractory NB [5]; however, the efficacy remains unsatisfactory. Therefore, it is essential to identify new drugs or treatment strategies that are more efficient and result in a lower incidence of toxic side effects.

Although arsenic trioxide (As_2_O_3_) is a drug that has been used in China for approximately 2000 years [6], its clinical application is limited due to its potential toxicity. In 1992, Hongde et al. [7]. demonstrated that As_2_O_3_ could be highly effective in the treatment of acute promyelocytic leukemia (APL), and increasing studies have shown that it can induce complete remission, even in patients with relapsed APL, at low doses and with minimal general toxicity [8]. In other studies, As_2_O_3_ has been shown to exert cytotoxic effects on a variety of tumors, such as lung cancer [9], nasopharyngeal carcinoma [10], osteosarcoma [11], neurogliocytoma [12], hepatoma [13], cervical cancer [14], cholangiocarcinoma [15], breast cancer [16], gastric cancer [17] and ovarian cancer [18]. These effects have been documented in vitro and in vivo. Recent studies have also shown that the growth and proliferation of a human NB cell line could be inhibited by As_2_O_3_ in a dose- and time-dependent manner and that N-myc mRNA levels could also be affected [19]. Moreover, As_2_O_3_ inhibited NB xenograft growth in nude mice [20]. Consequently, we hypothesized that As_2_O_3_ could feasibly be combined with existing chemotherapeutic modalities in order to establish a new alternative treatment strategy for high-risk NB patients [20]; however, to date, there have been no reports suggesting that As_2_O_3_ can be a valid alternative drug for NB clinical treatment.

NB is characterized by clinical heterogeneity and the complexity of genetic abnormalities [21]. Recently, molecular markers, including tyrosine kinase receptors (Trks), have been shown to be capable of transforming immortalized neurons. These proteins are involved in processes such as cell differentiation, proliferation, survival and death, treatment tolerance, invasion and genetic stability [22]. The expression of various members of the Trk family at different stages is clearly related to the prognosis and outcome of NB patients [23]. In NB, the Trk family of neurotrophin receptors, which includes TrkA, TrkB and TrkC, plays a prominent role in the biological behavior and efficacy of response-related gene regulation. The activation of TrkA-I isoforms and downstream signaling by nerve growth factor (NGF) ultimately leads to the apoptosis and differentiation of NB cells [24, 25]. Numerous studies have confirmed that elevated TrkA expression in NB tumor specimens is associated with a good prognosis [25]. TrkB is activated in NB via the endogenous expression of brain-derived neurotrophic factor (BDNF), and increased TrkB expression is an indicator of poor disease prognosis, since TrkB overexpression can lead to tumor cell growth and metastasis [26]. TrkC is a high-affinity receptor for neurotrophin-3 (NT-3), and exogenous NT-3 signals promote cellular apoptosis and differentiation [27]. In the current study, a favorable NB (stage 1/2/4S) was characterized by high levels of TrkA and TrkC expression, while the malignant (or aggressive) form (stage 3/4) was reported to overexpress TrkB [28].

Although As_2_O_3_ has been associated with complicated anticancer mechanisms, including the induction of tumor cell differentiation, the inhibition of tumor cell growth and the induction of apoptosis, its efficacy in different tumors remains unclear. In APL cells, As_2_O_3_ exerts anti-tumor effects by acting on the PML/RARA fusion protein [29]. Furthermore the anti-tumor effects of As_2_O_3_ have been associated with the activation of the caspase cascade, leading to apoptotic cell death and reduced expression of the Bcl-2 gene [30]. Gwak et al. [31]. demonstrated that As_2_O_3_ induced increases in p53 levels and decreases in cylinB1 levels combined with cell cycle arrest at the G2/M phase in U87MG and T98G glioblastoma cell lines. Qin et al. [32]. reported that As_2_O_3_ enhanced FOXO3a expression and p27^kip1^ transcription and that it may participate in the regulation of the growth of human hepatoma cells.

In the present study, immunohistochemical analysis was performed in order to examine the expression and distribution of the Trk family members during various stages of NB in children. We then examined the impact of As_2_O_3_ on the survival and cell cycle of NB cells. Finally, we used real-time RT-PCR in order to study changes in TrkA, TrkB and TrkC expression levels in NB cell lines following As_2_O_3_ treatment. The present study aimed to improve our understanding regarding the trend of TrkA, TrkB and TrkC gene expression in NB cell lines treated with As_2_O_3_. The findings provide experimental evidence regarding the exploration of the feasibility of As_2_O_3_ use in clinical NB treatment and can aid the assessment of chemotherapy efficacy and of long-term survival in NB children.

## Methods

### Specimens

A total of 12 NB samples from patients attending the Memorial Hospital of Sun Yat-sen University were evaluated. The diagnosis of NB in these patients was reviewed by our histopathologist, and the clinical staging was assessed using the International Neuroblastoma Staging System (INSS) [33]. The present study was approved by the Institutional Ethics Committee of the Memorial Hospital of the Sun Yat-sen University. Patients and their legal guardians signed informed consent agreements to permit the use of their tumor tissue in this research.

### Immunohistochemistry

Consecutive 3-µm sections were cut from every block, and an immunoperoxidase technique was implemented following antigen retrieval by either microwave treatment (95 °C), pepsin (DAKO; Agilent Technologies, Inc., Santa Clara, CA, USA) treatment for 20 min and/or citrate buffer (pH 6.0) treatment for 45 min. Following endogenous peroxidase blocking with 3% H_2_O_2_-methanol for 15 min, the specimens were rinsed with phosphate-buffered saline (PBS) for 3 times. Anti-TrkA, anti-TrkB and anti-TrkC antibodies (Abcam, Cambridge, UK) were diluted to 0.5 µg/mL and were used as primary antibodies. Following 12 h incubation at room temperature, the specimens were rinsed with PBS three times and treated for an hour at room temperature with the secondary antibody [peroxidase-conjugated anti-mouse or anti-rabbit antibody (Sigma-Aldrich; Merck KGaA, Darmstadt, Germany)] that was diluted to 0.5%. The probes were subsequently rinsed with PBS 3 times and staining was performed using diaminobenzidine (DAB) solution (DAKO). Following drying, the samples were counterstained with Meyer hematoxylin (Sigma-Aldrich). The immunostaining of all samples was conducted under the same conditions as the antibody reaction and DAB exposure. The evaluation of Trk expression was performed by observing 20 microscope fields at ×100 magnification (Santa Cruz Biotechnology, Inc., Dallas, TX, USA).

### Assessment of immunohistochemical staining

The staining intensity was scored on a scale of 1–3 as follows: 0 (absent), 1 (weak), 2 (moderate), or 3 (strong). The percentage of tumor cells that indicated positive staining was further assessed semi-quantitatively. The histochemical score (H-score) of immunoreactivity was obtained by multiplying the intensity and the percentage of positive cells, which yielded a final score of 0–300 [34]. The median expression level was selected as a cut-off value in order to classify the samples as positive or negative.

### Cell culture

Human SK-N-SH cells were obtained from the Medical School of the Sun Yat-sen University. The cells were maintained in Dulbecco’s modified Eagle’s medium/Nutrient Mixture F-12 Ham (DMEM/F12, 1:1 mixture) (Hyclone; GE Healthcare Life Sciences, Logan, UT, USA) supplemented with 10% heat-inactivated fetal bovine serum (Hyclone) at 37 °C in a 5% CO_2_ incubator.

### Experimental groups

To examine the effects of As_2_O_3_ on the cell cycle and the expression levels of Trk, SK-N-SH cells were divided into two treatment groups, and both groups were exposed to 4 µM As_2_O_3_ for 48 h. A medium sample devoid of As_2_O_3_ was used as a control. To determine the effects on gene expression, the cells were cultured at three different concentrations, namely, 1, 2 and/or 4 µM of As_2_O_3_ (10 mg, Beijing double-aigrettes pharmacy, China)., along with a control group for 24 and 48 h [2].

### Cell cycle distribution analysis

The cells were seeded into 6-well plates at a density of 2.5 × 10^5^ cells/well in 3 mL of medium and cultured overnight. Following serum starvation for 24 h, the cells were cultivated with half maximal inhibitory concentrations (IC50s) of As_2_O_3_ for 24 or 48 h in a 37 °C CO_2_ incubator. The cells were harvested by trypsinization, washed twice with PBS and fixed with cold 70% ethanol overnight. The samples were stained with propidium iodide (PI) solution containing 50 μg/mL RNase A and 0.1% Triton X-100. The cell cycle distribution was assessed using a flow cytometer (FCM-2012, BD, USA).

### Total RNA isolation

Following As_2_O_3_ treatment for 48 or 72 h, total RNA was isolated from SK-N-SH cell lines using the TRIzol reagent (Bio. Basic, Shanghai, China) according to the manufacturer’s protocol. The RNA was precipitated with isopropanol, washed with 75% ethanol, and dissolved in diethylpyrocarbonate (DEPC)-treated water. The integrity and purity of total RNA were assessed by DNA gel (1% agarose gel) electrophoresis and UV detection.

### Multiplex RT-PCR analysis

Following cDNA synthesis, PCR amplification was performed in order to examine the number of Trk transcripts. The assay was carried out using a Biometra T-gradient Thermoblock thermal cycler (Gottingen, Germany; Table 1). Multiplex RT-PCR was conducted in a final volume of 50 μL with 2× PCR-mix containing 200 μM dNTPs, 5% dimethyl sulfoxide (DMSO), 0.02 U HotStart Taq DNA polymerase (Qiagen GmbH, Hilden, Germany), and 50 pmol of each primer pair. The initial PCR cycle included activation of the polymerase at 95 °C for 5 min, which was followed by 35 cycles of PCR amplification (denaturation at 95 °C for 1 min, annealing at 55 °C for 1 min, and elongation at 72 °C for 1 min) and 1 cycle of elongation at 72 °C for 7 min prior to cooling at 4 °C. The primer sequences are listed in Table 1. The products from the multiplex RT-PCR were confirmed by specific Trks using individual PCRs, which were performed separately using the specific primers for each Trk. The conditions for the individual PCRs were identical to those of the multiplex reactions.

**Table 1:** Primer sequences and expected PCR product lengths in the multiplex PCR protocol

Gene	Size (bp)	Upstream primer (5′–3′)	Downstream primer (5′–3′)
TrkA	570	TCCAACACGGAGGCAATC	CCAGGACATCCAGGTAGACAG
TrkB	472	TCCGAGATTGGAGCCTAACAG	CCACCAGGATCAGTTCAGACA
TrkC	628	CAAGCCCACCCACTACAACA	GTGGGACTCACTTCGTCAAAC

### Statistical analysis

Each experiment was performed at least three times. The data are presented as the mean ± standard deviation (SD). All data were analyzed by Student’s *t* test using the SPSS 17.0 statistical software package (SPSS, Chicago, IL, USA); a P value lower than 0.05 (P < 0.05) was considered statistically significant.

## Results

### Specimens

The pediatric Sun Yat-sen Memorial Hospital database resulted in the identification of 12 patients diagnosed with NB; nine were stage 4 and three were stage 2. Since the three children with stage 2 disease had lower N-myc amplification, they were classified as intermediate risk, whereas the remaining of the subjects was classified as high risk according to the INSS criteria.

### Neurotrophin receptor expression in NB samples

**Fig. 1:**
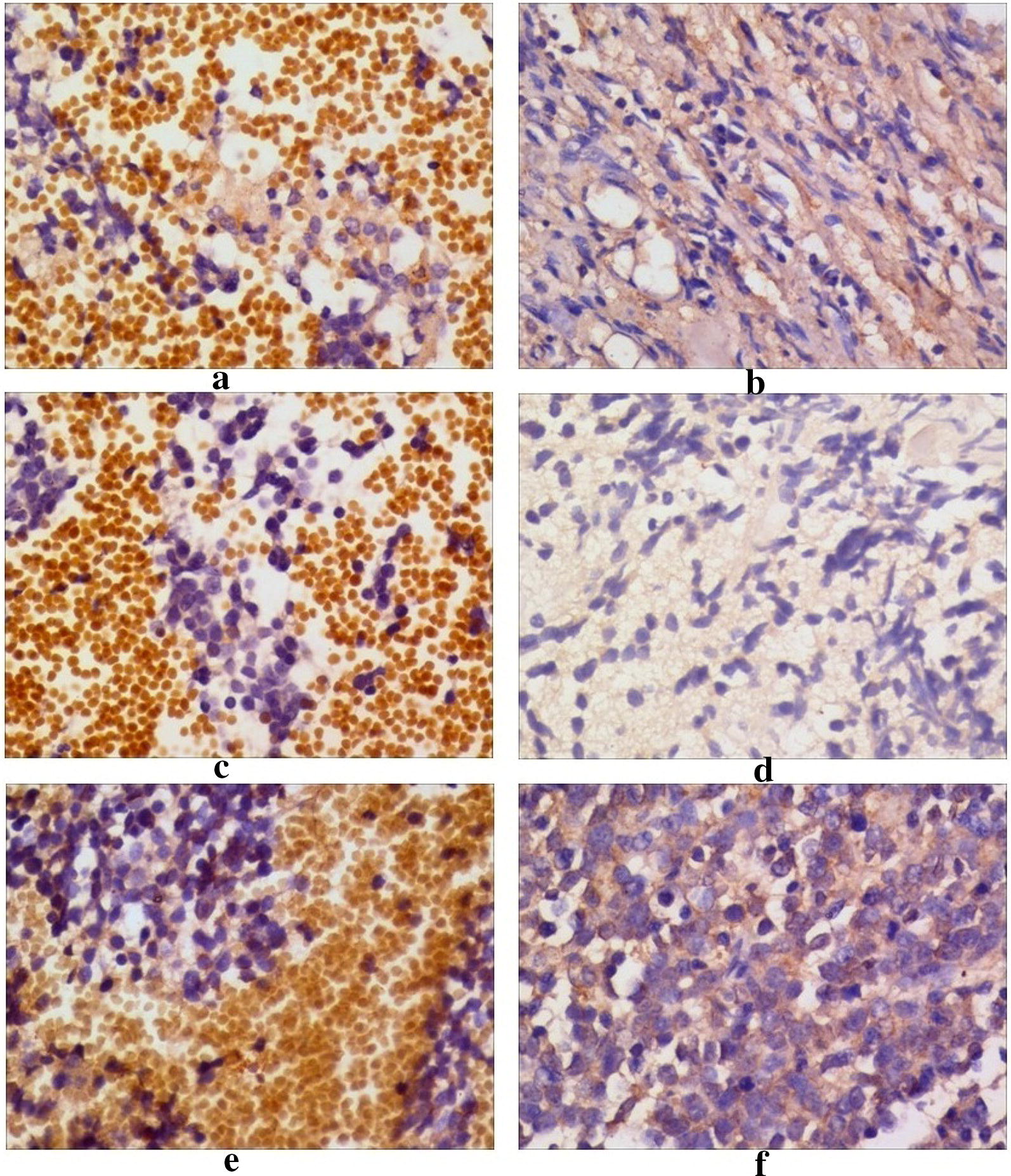
Trk expression in neuroblastoma pathological tissue. Immunohistochemical analyses of TrkA, TrkB TrkC expression. Immunoreactive labeling for TrkA (**a**), TrkB (**c**) and TrkC (**e**) was observed in the cytoplasm of NB cells. In the control cells, TrkA (**b**), TrkB (**d**) and TrkC (**f**) expression was observed. Original magnification ×200. Bar 100 µm

We performed immunohistochemical analyses of Trk expression in the 12 children with NB. The distribution of Trks was tissue-specific and correlated with the clinical heterogeneity of NB. TrkA expression was present in five (41.7%) tumors, with two (66.7%) stage 2 and three (33.3%) stage 4 tumors showing TrkA expression. A total of 11 (91.7%) tumors expressed TrkB, with two (66.7%) stage 2 and nine (100%) stage 4 tumors. Four (33.3%) tumors exhibited TrkC expression and were divided into two (66.7%) stage 2 and two (22.2%) stage 4 tumors. It is interesting to note that both TrkA (66.7%) and TrkC (66.7%) were strongly co-expressed in stage 2 samples, although they indicated low co-expression in stage 4 samples. Furthermore, TrkB (100%) was highly expressed in advanced-stage disease (stage 4), whereas it was expressed to a relatively lower extent (66.7%) in early-stage NB (stage 2) (Fig. 1).

### As_2_O_3_ induces G2/M phase arrest

**Fig. 2:**
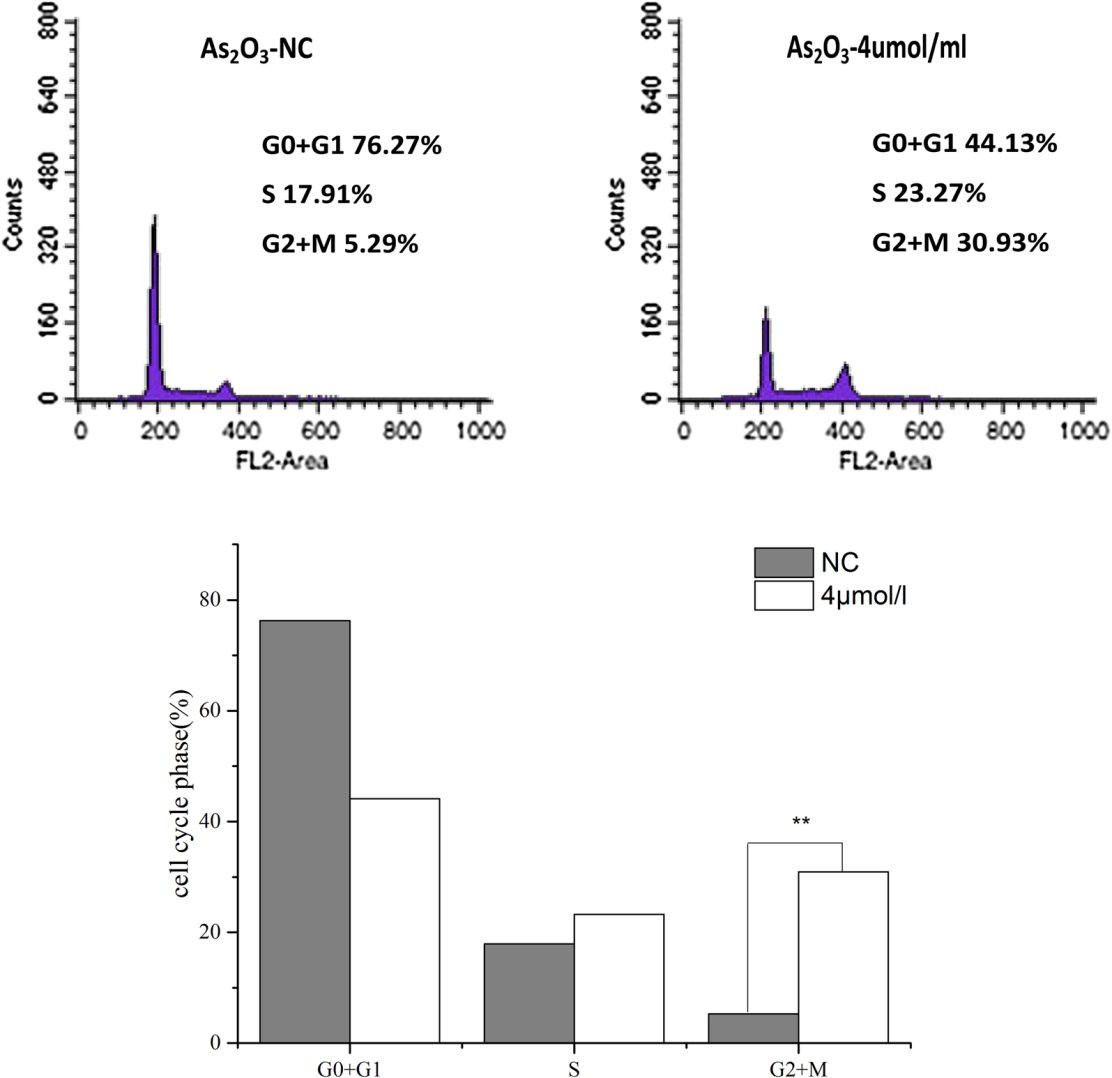
As_2_O_3_-induced S-G2/M arrest in NB cells. The effect of As_2_O_3_ on cell cycle distribution in SK-N-SH cells was investigated by flow cytometry following 48 h of treatment. The percentages of the cells in the various stages of the cell cycle were quantified

Different chemotherapeutic agents have various mechanisms by which they affect cell cycle phases, including the blockade of G_1_-S and G2/M checkpoints, the proliferative arrest, the onset of DNA repair and the activation of programmed cell death. We analyzed the cell cycle distribution of As_2_O_3_-treated SK-N-SH cells by flow cytometry. The cells were treated with 4 µM of As_2_O_3_ for 48 h, and the results of the cell cycle analyses are shown in Fig. 2. The percentage of G_0_/G_1_-phase cells decreased from 76.27% in control cells to 44.13% in cells treated with 4 µM As_2_O_3_. Concomitantly, the percentage of G2/M phase cells in the group treated with 4 μM As_2_O_3_ (30.93%) was significantly higher (P < 0.01) than that noted in the control group (5.29%). These data suggested that As_2_O_3_ induced apoptosis of SK-N-SH cells following cell cycle arrest at the G2/M phase (Fig. 2).

### As_2_O_3_ affects Trk mRNA expression in SK-N-SH cells

**Fig. 3:**
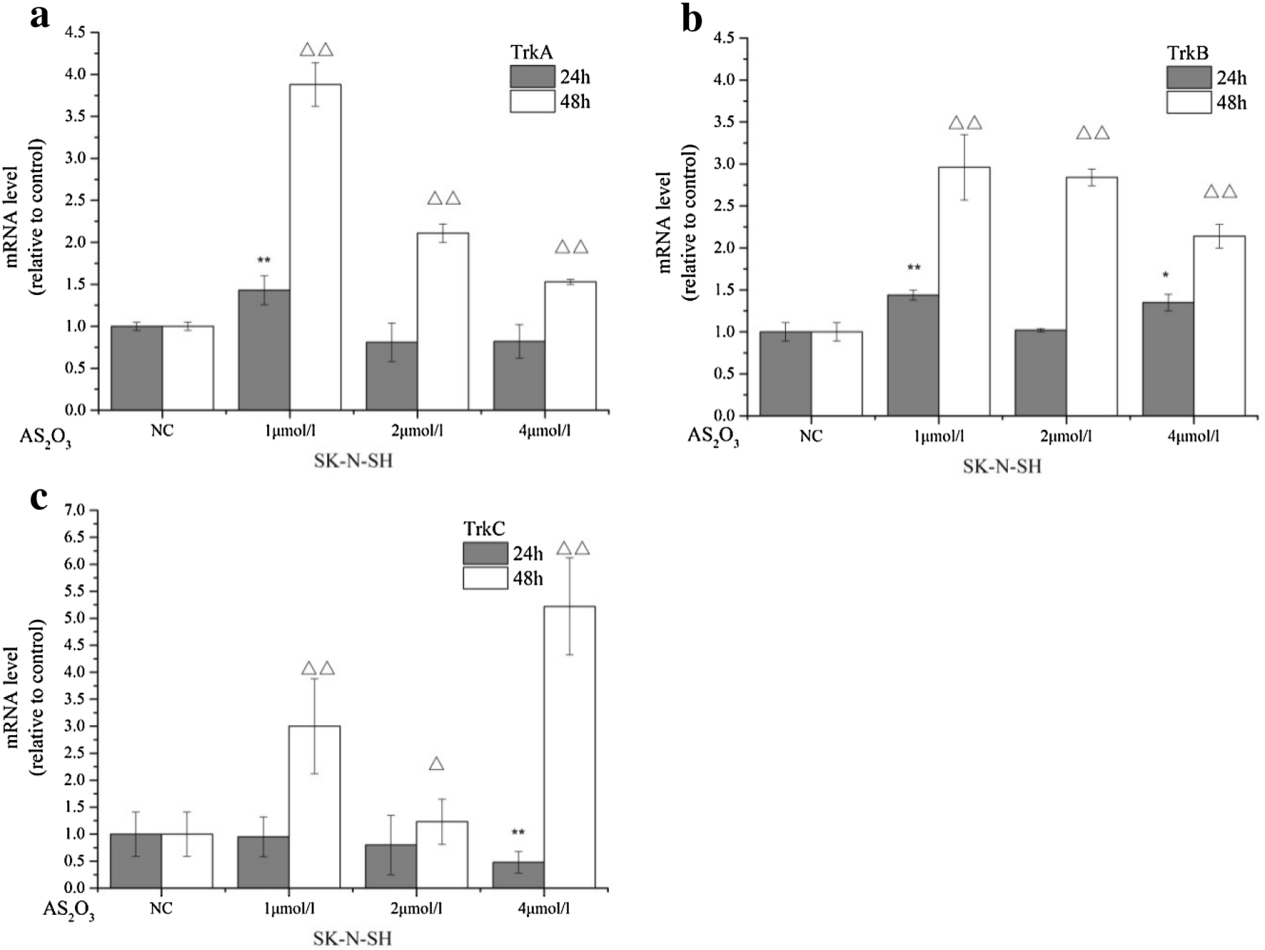
As_2_O_3_ enhances the expression of *Trk* genes in SH-N-SH Cells. RT-PCR was performed to analyze the mRNA expression levels of *Trk* genes. RT-PCR was performed on SK-N-SH cells that were treated with 0, 1, 2 or 4 µM As_2_O_3_ for 24 or 48 h. The data shown were compiled from three independent experiments

The mRNA expression levels of TrkA, TrkB and TrkC were subsequently analyzed by RT-PCR (Fig. 3). TrkA, TrkB and TrkC were all expressed in SK-N-SH cells. TrkA levels were significantly increased in cells treated with 1 µM As_2_O_3_ for 48 h compared with those treated for 24 h (P < 0.01). In contrast to the 1 μM dose of As_2_O_3_, TrkA was downregulated following 2 and 4 µM As_2_O_3_ treatment. This trend was increased at the 48-h period compared with the 24-h period of treatment (Fig. 3a). Additional results demonstrated that the TrkB gene was expressed mainly in stage 3 and 4 malignant NB cells, which is consistent with previous studies [3]. TrkB expression in SK-N-SH cells increased 2.14-fold following treatment with 1 μM of As_2_O_3_ at 48 h, whereas 4 μM treatment caused a 2.96-fold increase in expression (P < 0.01) compared with that of the control group. As_2_O_3_ increased TrkB mRNA expression in a dose-dependent manner at 24 and 48 h. In addition, RT-PCR analyses indicated that TrkC expression levels were significantly higher in SK-N-SH cells treated with 4 µM As_2_O_3_ for 48 h (5.22 ± 0.90) compared with untreated cells (1.00 ± 0.41, P < 0.01) (Fig. 3b).

## Discussion

The possible prognostic factors related to the clinical behavior of NB have been identified by recent studies [35]. Trks, with their respective neurotrophin ligands, have been strongly correlated with clinical outcomes [36]. The function of these receptors is associated with the survival and differentiation of neurons in the nervous system [37]. Immunohistochemical staining for Trk expression in NB tumors demonstrated that TrkA and TrkC were expressed more prominently in stage 2 tissues. Moreover, TrkB was expressed in all stage 4 tissues, and only in some of the earlier stage tissues. Unfortunately, the present study used a low sample number and did not permit the assessment of the statistical significance of Trk distribution in tissues from different stages. Similarly, Hoehner et al. [38] assessed Trk expression at the mRNA level by real-time PCR assays and reported that TrkA and TrkC expression correlated with favorable outcomes and early stage tumor progression. TrkB was expressed in homogenates of late-stage NB patients with poor prognoses. The authors of this study postulated that in low-stage NB, TrkA and/or TrkC responsiveness may play a role in persistent tumor growth and/or regression [39]. The findings indicated that, TrkA, TrkB and TrkC should be considered new prognostic markers for NB.

As_2_O_3_ has been effectively used as a therapeutic agent to treat solid tumors and acts through the induction of cell cycle arrest or apoptosis [40]. Suppression of cell growth by As_2_O_3_ can be explained in part by its capacity to affect cell cycle distribution. Ruggeri et al. [41]. reported that low As_2_O_3_ concentrations (4 µM) induced rapid induction of apoptosis in NB cells at a rate of more than 50% in less than 16 h. Moreover, kinase activity significantly increased in those cells treated with 4 µM As_2_O_3_. The present study demonstrated that the treatment of SK-N-SH cells with 4 µM As_2_O_3_ for 48 h induced arrest at the G2/M phase. Our preliminary results (data not shown here) showed that the apoptosis rates of SK-N-SH cells during different time periods of As_2_O_3_ treatment were higher than those of the control cells. A similar study was performed by Woo et al. [42],who used SH-SY5Y and SK-N-AS NB cells co-cultured with As_2_O_3_. Both cell lines were arrested at the S-G2/M phase and this effect was associated with increased cyclin B expression and CDK1 activity [42, 43]. Other studies observed that the effect of As_2_O_3_ on cell cycle progression in NB cells may be related to the p53 status [44] and the micro-protein polymerization. These findings suggested that As_2_O_3_ could arrest the cell cycle by modulating the expression and/or activity of several key G2/M regulatory proteins [45]. Furthermore, the activation of caspases occurred solely in As_2_O_3_-induced mitotic cells only, as opposed to interphase cells [46], suggesting that As_2_O_3_ has a higher cytotoxicity against NB cells that are arrested at the G2/M phase. In addition, the capacity of enhancing treatment outcomes by arresting cells during the S phase of the cell cycle via the combination of As_2_O_3_ and chemotherapy should be considered.

Chemotherapeutics arrest the tumor cell cycle and further influence the expression of certain genes that closely correlate with cancer stage and prognosis [47]. The present study is the first to demonstrate that As_2_O_3_ can influence TrkA, TrkB and TrkC expression in a time- and dose-dependent manner. We further demonstrated that increasing As_2_O_3_ concentrations caused significant upregulation of TrkA and TrkC expression levels. This observation was more obvious at the 48 h compared with the 24 h time point. In human NBs, TrkA is involved in the inhibition of cell growth, the induction of apoptosis, the inhibition of angiogenesis and the determination of either apoptosis and/or differentiation [48]. The selective synthetic TrkA tyrosine kinase inhibitor 5C3 directly binds to the extracellular domain of TrkA and promotes receptor phosphorylation and activation of the PI3K pathway, which leads to NB cell apoptosis [49]. One report indicated that TrkC expression in tumors is often associated with favorable prognosis, suggesting that TrkC acts as a tumor suppressor and/or as a dependence receptor, regulating neuronal survival [39]. High levels of TrkA and TrkC expression in NB patients were associated with favorable prognosis; therefore, the data of the present study suggest that any treatment of NB at stages 1, 2 and 4S should maintain high levels of TrkA and TrkC expression. Furthermore, the therapeutic concentration and the duration of As_2_O_3_ treatment should be increased.

The receptor TrkB is preferentially expressed in NB patients who have a poor prognosis and confers invasive and metastatic potential to tumor cells in addition to enhancing therapeutic resistance [50]. We further demonstrated that TrkB expression in SK-N-SH cells was inversely proportional to the concentration of As_2_O_3_, with the level of TrkB in the group treated with 4 µM for 48 h being significantly less than that in the control group. Increased TrkB expression is believed to be associated with aggressive NB. Zhang et al. [51] demonstrated that TrkB-expressing NB cells can block cisplatin-induced cell death. Cimmino et al. [52] reported that the BDNF-mediated activation of TrkB enhanced the invasiveness and migration of NB cells in vitro, an effect that could be impaired by the transient transfection of a Gal-1-specific siRNA or a neutralizing antibody directed against Gal-1. Taken together, the data suggest that TrkB downregulation is a potential therapeutic target for refractory or relapsed NB. Our findings further suggested the use of As_2_O_3_ for the treatment of stage 3 and stage 4 NB, which indicate high levels of TrkB expression and poor prognoses; an appropriate concentration and treatment time for As_2_O_3_ remains to be determined.

## Abbreviations

As_2_O_3_: arsenic trioxide
NB: neuroblastoma
Trks: tyrosine kinase receptors
auto-HSCT: auto-hematopoietic stem cell transplantation
APL: acute promyelocytic leukemia
NGF: nerve growth factor
BDNF: brain-derived neurotrophic factor
NT-3: neurotrophin-3
INSS: International Neuroblastoma Staging System
PBS: phosphate-buffered saline
IC50s: 50% inhibitory concentrations
PI: propidium iodide
DMSO: dimethyl sulfoxide
